# Modular versus non-modular humeral stems in shoulder arthroplasty for proximal humerus fracture: a multicenter retrospective cohort study

**DOI:** 10.1016/j.jor.2026.04.009

**Published:** 2026-04-30

**Authors:** Aaron M. Atlas, Shawn J. Geffken, Matthew T. Geiselmann, Benjamin Hershfeld, Anthony Modica, Randy M. Cohn

**Affiliations:** a Northwell Health, New Hyde Park, NY, USA; b Department of Orthopaedic Surgery, Northwell Health – Huntington Hospital, 270 Park Ave, Huntington, NY, 11743, USA; c Donald and Barbara Zucker School of Medicine at Hofstra/Northwell, 500 Hofstra Blvd, Hempstead, NY, 11549, USA

**Keywords:** Shoulder arthroplasty, Proximal humerus fracture, Modular humeral stem, Reverse shoulder arthroplasty, Patient outcomes, Operative time

## Abstract

**Purpose:**

Evidence directly comparing modular and non-modular humeral stems in shoulder arthroplasty for proximal humerus fractures (PHF) remains limited.

**Objective:**

This study aimed to compare perioperative outcomes, short-term complications, and patient-reported outcomes following shoulder arthroplasty for PHF using modular versus non-modular humeral stems.

**Methods:**

Adult patients who underwent shoulder arthroplasty for PHF between January 2014 and July 2023 across a multicenter health system were retrospectively identified using billing data and confirmed by electronic medical record review. Patients were grouped according to humeral stem design as modular or non-modular. Outcomes included operative time, length of stay (LOS), discharge disposition, 1-year adverse events, postoperative American Shoulder and Elbow Surgeons (ASES) score, and visual analog scale (VAS) pain score. Continuous and categorical variables were compared between groups, with statistical significance set at p < 0.05.

**Results:**

A total of 319 shoulder arthroplasties in 318 patients were included, including 89 modular and 230 non-modular cases. Modular stems were associated with shorter operative time (115.0 vs. 149.0 min, p < 0.001), shorter LOS (1.9 vs. 3.3 days, p < 0.001), and higher rates of discharge to home (82.0% vs. 67.4%, p = 0.009). Postoperative ASES scores were significantly higher in the modular group (72.4 vs. 63.1, p = 0.02), whereas VAS pain scores were similar between groups (p = 0.1). Most short-term adverse events were comparable, although prosthetic dislocation was more frequent in the modular group (3.4% vs. 0.4%, p = 0.04).

**Conclusion:**

Modular humeral stems were associated with shorter operative time, shorter hospital stay, and higher postoperative functional scores after shoulder arthroplasty for PHF. Most short-term complications were similar between groups, although prosthetic dislocation was significantly more common with modular stems.

## 1 Introduction

Proximal humerus fractures (PHF) account for 5% to 6% of all adult fractures and occur predominantly in older adults after low-energy falls.^1^^,^^2^ Their incidence has continued to rise, reaching 105 per 100,000 person-years in 2019, with age-adjusted incidence exceeding 400 per 100,000 in patients aged 80 years and older.^3^ Management remains challenging, particularly for displaced 3-part and 4-part fractures in osteoporotic bone, where tuberosity displacement and distorted anatomy can limit fixation and recovery.^4^ Although many PHFs are treated nonoperatively, shoulder arthroplasty has an established role in unreconstructible or highly complex patterns, especially in older patients.^5^ Reverse shoulder arthroplasty (RSA) has demonstrated superior functional outcomes versus plate fixation for displaced 3-part and 4-part fractures in elderly patients and lower complication and reoperation rates than hemiarthroplasty in systematic review data.^6^, ^7^, ^8^ Utilization trends reflect this shift, with prior studies reporting a marked increase in RSA use for PHF over the last decade.^9^ Recent data have also shown complication rates broadly comparable to elective indications.^10^^,^^11^

With the expanding role of arthroplasty for fracture, humeral stem design has become an important determinant of reconstruction. In fracture arthroplasty, outcomes depend on restoration of humeral height and version, secure tuberosity fixation, graft incorporation, and appropriate soft-tissue tensioning, all of which influence stability and postoperative function.^12^ Fracture-specific stems were developed to address these technical demands and to facilitate tuberosity repair, graft incorporation, and fracture reconstruction in the setting of distorted proximal humeral anatomy. Comparative and meta-analytic studies have shown that fracture-specific stems are associated with improved functional scores and higher tuberosity healing rates than conventional stems in shoulder arthroplasty for PHF.^13^, ^14^, ^15^ This is clinically relevant because anatomic tuberosity healing remains associated with improved range of motion and functional outcomes after fracture arthroplasty, including RSA.^16^^,^^17^

Modularity may offer additional advantages within this broader evolution in stem design. Modular humeral stems permit intraoperative adjustment of version, height, offset, and construct configuration, which may be particularly useful in fracture settings where anatomic landmarks are distorted or absent.^18^ Indirect evidence from revision and conversion arthroplasty suggests that modular platforms can reduce operative time, blood loss, and intraoperative morbidity when the stem can be retained.^19^^,^^20^ However, modularity may also introduce disadvantages, including malposition-related instability, junction-related complications, and higher loosening or revision risk in some settings.^21^, ^22^, ^23^.

Despite growing use of both fracture-specific and modular implants, direct comparative evidence on modular versus non-modular humeral stems in PHF arthroplasty remains limited. Much of the literature compares fracture-specific with conventional stems, which does not isolate modularity from other design features, or is drawn from elective and revision settings that may not generalize to acute fracture care.^15^^,^^24^ The purpose of this study was to compare short-term complications, perioperative efficiency, and available patient-reported outcomes after shoulder arthroplasty for PHF using modular versus non-modular humeral stems. We hypothesized that modular stems would demonstrate comparable short-term safety, with potential differences in operative efficiency and functional outcome.

## 2 Materials and methods

### 2.1 Study design and setting

This retrospective cohort study evaluated adult patients (age ≥18) who underwent shoulder arthroplasty for PHF across a large multicenter health system between January 2014 and July 2023. Institutional Review Board approval was obtained (IRB: 23-0312), and informed consent was waived.

### 2.2 Patient identification and eligibility

Potential cases were identified using systemwide billing data and Current Procedural Terminology (CPT) codes 234724 for total shoulder arthroplasty (TSA) or RSA and 23470 for partial shoulder arthroplasty or hemiarthroplasty (HA). The electronic medical record (EMR) was then reviewed to confirm PHF as the indication for surgery. Patients undergoing arthroplasty for acute fracture, malunion, or nonunion were eligible for inclusion. Exclusion criteria were infection, tumor, revision arthroplasty or hardware removal, inability to determine implant type, and less than 1 year of postoperative follow-up.

### 2.3 Exposure definition, clinical, fracture, and operative variables

Patients were categorized according to humeral stem design as modular or non-modular based on implant information documented in the operative record. Implant selection, arthroplasty type, fixation strategy, and intraoperative management were determined by the treating surgeon. Specific decision criteria were not standardized across surgeons, and factors such as surgeon-assessed bone quality or osteoporosis severity were not consistently documented and therefore could not be analyzed. Demographic and baseline clinical variables included age, sex, body mass index (BMI), American Society of Anesthesiologists (ASA) class, and medical comorbidities. Charlson Comorbidity Index (CCI) scores were calculated from the documented medical history. Fracture characteristics were assessed independently by two reviewers using preoperative radiographs and computed tomography when available. Recorded fracture variables included Neer classification, head-splitting fracture pattern, fracture-dislocation, and polytrauma status. Disagreements in fracture classification were resolved by a third independent reviewer. Operative records were reviewed to determine surgical indication, arthroplasty type, implant characteristics, fixation method, and intraoperative complications. Additional perioperative variables included time from injury to surgery, operative time, length of stay (LOS), and discharge disposition. Surgeon fellowship training was verified through review of publicly available institutional profiles.

### 2.4 Outcomes

Study outcomes included perioperative variables, short-term adverse events, and available patient-reported outcomes. Perioperative outcomes included operative time, LOS, and discharge disposition. Short-term adverse events were identified through EMR review within 1 year of surgery and included intraoperative fracture, postoperative periprosthetic fracture, wound complication, infection, prosthetic dislocation, deep vein thrombosis (DVT), return to operating room (RTOR), revision shoulder arthroplasty, return to the emergency department (ED), and all-cause readmission. Available patient-reported outcomes included postoperative American Shoulder and Elbow Surgeons (ASES) and visual analog scale (VAS) pain scores. ASES scores were obtained by patient contact, and VAS pain scores were recorded when available in the medical record. Patient-reported outcomes were analyzed on an available-case basis.

### 2.5 Statistical analysis

Patients were compared between modular and non-modular stem cohorts. Continuous variables were summarized as means with standard deviations and compared using Welch's t-tests. Categorical variables were summarized as counts with percentages and compared using chi-square tests. All tests were 2-sided, and statistical significance was defined as p < 0.05. Statistical analyses were performed using Jamovi software (version 2.3.28; Sydney, Australia).

## 3 Results

### 3.1 Cohort and baseline characteristics

A total of 319 shoulder arthroplasties for PHF were included in 318 patients, including one bilateral case. Overall, 27.9% of cases were treated with modular stems and 72.1% with non-modular stems. Mean age was 71.2 ± 10.0 years, mean BMI was 29.0 ± 6.6 kg/m^2^, and mean CCI was 3.4 ± 1.6. The cohort was predominantly female (76.5%), and the most common ASA class was III (54.0%). Baseline patient characteristics were similar between cohorts. There were no significant differences in age, BMI, CCI, sex distribution, or ASA class distribution between the modular and non-modular groups (all p > 0.05) (Table 1).

**Table 1:** Patient demographics.

Variable	Total (N = 319)	Modular (n = 89; 27.9%)	Non-Modular (n = 230; 72.1%)	p-value
**Age (yr)**	71.2 ± 10.0	71.2 ± 8.2	71.3 ± 10.7	1.0
**BMI**	29.0 ± 6.6	29.3 ± 7.1	28.9 ± 6.5	0.6
**CCI**	3.4 ± 1.6	3.4 ± 1.5	3.4 ± 1.7	0.9
**Gender**				0.6
Male	75 (23.5%)	19 (21.3%)	56 (24.3%)	
Female	244 (76.5%)	70 (78.7%)	174 (75.7%)	
**ASA Class**				0.4
I	5 (1.7%)	2 (2.5%)	3 (1.3%)	
II	127 (42.1%)	32 (40.5%)	95 (42.6%)	
III	163 (54.0%)	45 (60.0%)	118 (52.9%)	
IV	7 (2.3%)	0 (0.0%)	7 (3.1%)	

**Notes:** Continuous data are presented as mean ± standard deviation; categorical data are presented as count (percentage). P values were calculated using Welch's *t*-test for continuous variables and chi-square tests for categorical variables. A p value < 0.05 was considered statistically significant.

**Abbreviations:** ASA = American Society of Anesthesiologists score; BMI = Body Mass Index; CCI = Charlson Comorbidity Index.

### 3.2 Injury and operative characteristics

Overall, 75.5% of procedures were RSAs, 24.1% were HAs, and 0.3% were TSAs. Procedure distribution differed between cohorts (p = 0.02), with RSA performed more frequently in the modular cohort and HA performed more frequently in the non-modular cohort (Table 2). Percentages for fracture characteristics reflect available data for each variable.

**Table 2:** Injury and operative characteristics.

Variable	Total	Modular	Non-Modular	p-value
Fracture Parts				
II	41 (13.3%)	22 (25.3%)	19 (8.6%)	<0.001
III	66 (21.4%)	27 (31.0%)	39 (17.6%)	0.009
IV	202 (65.4%)	38 (43.7%)	164 (73.9%)	<0.001
**Fx-Dislocation**	76 (24.6%)	10 (11.8%)	66 (29.3%)	0.001
**Head-Splitting Fx**	28 (9.1%)	5 (5.9%)	23 (10.3%)	0.23
**Polytrauma**	38 (11.9%)	7 (8.0%)	31 (13.6%)	0.17
**Mean Time From Injury to OR (days)**	66.6 ± 160.3	104.0 ± 194.6	52.2 ± 142.6	0.01
**Procedure**				0.02
HA	77 (24.1%)	12 (13.5%)	65 (28.3%)	
RSA	241 (75.5%)	77 (86.5%)	164 (71.3%)	
TSA	1 (0.3%)	0 (0.0%)	1 (0.4%)	
**Subscapularis Repaired**	251 (78.7%)	62 (69.7%)	189 (79.6%)	0.01
**Surgical Time (minutes)**	140 ± 52.7	115 ± 42.3	149 ± 53.1	<0.001
**Fixation**				<0.001
Press-fit	139 (43.6%)	72 (80.9%)	67 (29.1%)	
Cemented	180 (56.4%)	17 (19.1%)	163 (70.9%)	
**LOS (days)**	2.9 ± 4.0	1.9 ± 2.3	3.3 ± 4.4	<0.001
**Discharge Disposition**				0.009
Home	228 (71.5%)	73 (82.0%)	155 (67.4%)	
Rehab	91 (28.5%)	16 (18.0%)	75 (32.6%)	

**Notes:** Continuous data are presented as mean ± standard deviation; categorical data are presented as count (percentage). P values were calculated using Welch's *t*-test for continuous variables and chi-square tests for categorical variables. A p value < 0.05 was considered statistically significant.

**Abbreviations:** Fx = Fracture; LOS = Length of Stay; OR = Operating Room; HA = Hemiarthroplasty; RSA = Reverse Shoulder Arthroplasty; TSA = Total Shoulder Arthroplasty.

Modular stems were used more frequently in 2-part fractures (25.3% vs. 8.6%, p < 0.001) and 3-part fractures (31.0% vs. 17.6%, p = 0.009), whereas non-modular stems were used more frequently in 4-part fractures (73.9% vs. 43.7%, p < 0.001) and fracture-dislocations (29.3% vs. 11.8%, p = 0.001). Rates of head-splitting fracture pattern and polytrauma were similar between groups (both p > 0.05). Mean time from injury to surgery was longer in the modular cohort (104.0 ± 194.6 vs 52.2 ± 142.6 days, p = 0.01).

Operative characteristics also differed between cohorts. Subscapularis repair was less common in the modular cohort (69.7% vs. 79.6%, p = 0.01). Cemented fixation was more common in the non-modular cohort (70.9% vs. 19.1%, p < 0.001), whereas press-fit fixation was more common in the modular cohort (80.9% vs. 29.1%, p < 0.001). Mean operative time was shorter in the modular cohort (115.0 ± 42.3 vs. 149.0 ± 53.1 min, p < 0.001), as was mean LOS (1.9 ± 2.3 vs. 3.3 ± 4.4 days, p < 0.001). Discharge to home was also more common in the modular cohort (82.0% vs. 67.4%, p = 0.009) (Table 2).

### 3.3 Patient-reported outcomes and short-term adverse events

The overall ASES response rate was 27.0% (86/319), including 38.2% in the modular cohort (34/89) and 22.6% in the non-modular cohort (52/230). Mean postoperative ASES score was higher in the modular cohort (72.4 ± 15.6 vs. 63.1 ± 20.1, p = 0.02). Mean VAS pain score did not differ significantly between groups (2.2 ± 2.1 vs. 3.1 ± 2.8, p = 0.1) (Table 3).

**Table 3:** Operative outcomes and complication rates.

Variable	Total	Modular	Non-Modular	p-value
ASES Score Response Rate	86 (27.0%)	34 (38.2%)	52 (22.6%)	—
ASES Score	66.8 ± 18.9	72.4 ± 15.6	63.1 ± 20.1	0.02
VAS Score	2.8 ± 2.5	2.2 ± 2.1	3.1 ± 2.8	0.1
RTED	68 (21.3%)	20 (22.5%)	48 (20.9%)	0.8
Readmission	45 (14.1%)	13 (14.6%)	32 (13.9%)	0.9
RTOR	36 (11.3%)	12 (13.5%)	24 (10.4%)	0.4
Revision	11 (3.4%)	5 (5.6%)	6 (2.6%)	0.2
Intraoperative Fx	5 (1.6%)	2 (2.2%)	3 (1.3%)	0.5
Any Postoperative Complications	19 (6.0%)	8 (9.0%)	11 (4.8%)	0.2
Postoperative Periprosthetic Fx	6 (1.9%)	0 (0.0%)	6 (2.6%)	0.1
Infection	5 (1.6%)	2 (2.2%)	3 (1.3%)	0.5
Wound Complication	4 (1.3%)	2 (2.2%)	2 (0.9%)	0.3
Prosthetic Dislocations	4 (1.3%)	3 (3.4%)	1 (0.4%)	0.04
DVT	6 (1.9%)	3 (3.4%)	3 (1.3%)	0.2

**Notes:** Data are presented as mean ± standard deviation or n (%). Patient-reported outcomes were analyzed on an available-case basis. Short-term adverse events were assessed within 1 year postoperatively. P values were calculated using Welch's *t*-test for continuous variables and chi-square tests for categorical variables. A p value < 0.05 was considered statistically significant.

**Abbreviations:** ASES = American Shoulder and Elbow Surgeons; DVT = Deep Vein Thrombosis; Fx = Fracture; RTED = Return to Emergency Department; RTOR = Return to Operating Room; VAS = Visual Analogue Scale.

**Fig. 1:**
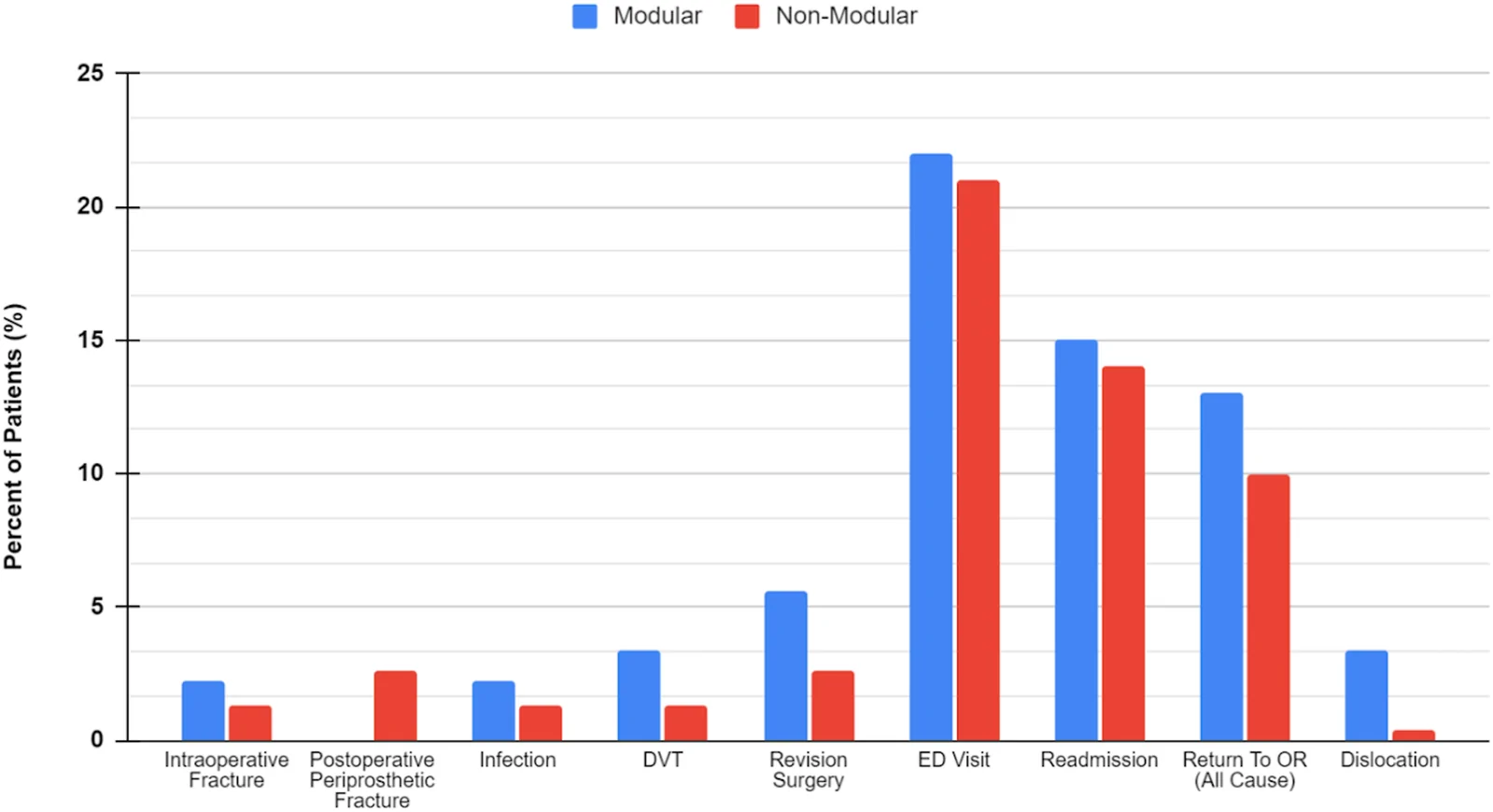
One-year adverse events by humeral stem design after shoulder arthroplasty for proximal humerus fracture. Bar graph comparing the percentage of patients with each recorded adverse event in the modular (blue) and non-modular (red) humeral stem cohorts within 1 year after surgery. Abbreviations: DVT = Deep Vein Thrombosis; ED = Emergency Department; OR = Operating Room.

Most short-term adverse events were similar between cohorts including return to ED, readmission, RTOR, revision shoulder arthroplasty, intraoperative fracture, any postoperative complication, postoperative periprosthetic fracture, infection, wound complication, and DVT (all p > 0.05). Prosthetic dislocation was the only adverse event that differed significantly and occurred more frequently in the modular cohort (3/89 [3.4%] vs. 1/230 [0.4%], p = 0.04) (Table 3, Fig. 1).

## 4 Discussion

In this multicenter retrospective cohort of shoulder arthroplasty for PHF, modular humeral stems were associated with a similar overall short-term adverse event profile, shorter operative time, shorter LOS, higher rates of discharge to home, and higher available ASES scores compared with non-modular stems. Prosthetic dislocation was the only adverse event that differed significantly and occurred more frequently in the modular cohort. These findings are clinically relevant because arthroplasty for PHF is generally a more complex episode of care than elective shoulder arthroplasty, and operative efficiency, LOS, discharge disposition, and shoulder-specific function all affect recovery and resource utilization.^25^ The difference in ASES despite similar VAS pain scores is also plausible, as shoulder-specific measures may capture functional recovery better than pain scores alone after RSA for PHF.^26^

The overall safety findings are reassuring. Most short-term adverse events, including RTOR, revision arthroplasty, infection, wound complication, fracture, DVT, ED return, and readmission, were similar between cohorts. This is consistent with contemporary literature suggesting that complication rates after PHF arthroplasty have improved over time and that large short-term safety differences across implant designs are not consistently observed. In a national surgical quality improvement database analysis of 5889 PHF operations, RSA use increased from 4% in 2007 to 34% in 2018, while HA declined from 50% to 9%, and minor complication rates decreased over time across treatment modalities.^27^ Registry data likewise suggest that fracture arthroplasty carries a higher early revision risk than arthroplasty for degenerative conditions, but do not clearly identify stem design as a dominant driver of failure. In the Dutch Arthroplasty Register, which included 10,857 RSAs, fracture indications were associated with a higher 1-year revision risk than degenerative indications, with a hazard ratio of 2.50, yet no difference was seen between conventional and fracture-specific stems within the fracture cohort.^28^ These data support modular stems as a reasonable option in fracture arthroplasty, but not as proven superior in overall safety.

The efficiency and functional findings require more careful interpretation. The shorter operative time, shorter LOS, and higher available ASES scores with modular stems are directionally consistent with the added intraoperative flexibility modular stems provide during fracture reconstruction.^18^ In a direct comparative study of modular versus non-modular stems in fracture RSA, modular stems were associated with higher ASES scores, 76.35 versus 65.58, whereas non-modular stems demonstrated better abduction and better external rotation**,** suggesting that functional advantages may differ depending on the outcome assessed rather than uniformly favoring one design. Additionally, operative time in that series was longer with modular stems.^29^ The difference between that study and the present findings likely reflects variation in implant construct, procedure mix, fracture pattern, surgeon preference, or treatment setting rather than a true contradiction. Accordingly, the favorable efficiency and functional signals observed should be interpreted as associations rather than evidence that modularity alone improves these outcomes.

The higher dislocation rate in the modular cohort deserves separate emphasis. Although the absolute number of events was small, dislocation was the only adverse event that differed significantly between groups, and this finding is clinically important because postoperative stability is a central goal of arthroplasty. Instability after RSA can lead to recurrent events, reoperation, and worse function. In a multicenter study of 6621 RSAs, the overall dislocation rate was 2.1%, including 1.6% in primary cases and 6.5% in revision cases, and important predictors included fracture nonunion and absence of subscapularis repair.^30^ A meta-analysis further reported lower instability with subscapularis repair in medialized constructs, 0.8% versus 4.2%.^31^ These data are relevant because subscapularis repair was less common in the modular cohort in the present study. However, subscapularis management likely does not fully explain the finding. PHF itself is an instability-prone indication after RSA, and instability is also influenced by construct tension, glenoid-sided positioning, and technical variables not captured in the present study.^32^^,^^33^ Revision literature further suggests that modular platforms do not eliminate the consequences of malposition, as stem exchange was still required in more than 30% of modular revision cases in one systematic review.^22^ The dislocation signal should therefore be interpreted cautiously as an important association rather than proof that modularity independently increases instability risk.

This study has several limitations. First, its retrospective, nonrandomized design introduces the possibility of confounding by indication. The cohorts differed in fracture pattern, arthroplasty type, fixation strategy, time to surgery, and subscapularis management, all of which may have influenced efficiency, discharge disposition, patient-reported outcomes, and instability independent of stem modularity. Second, patient-reported outcome measure availability was limited and analyzed on an available-case basis, which may have introduced response bias. Third, radiographic and construct-specific variables such as tuberosity healing, stem position, version, and glenoid-sided parameters were not assessed, limiting mechanistic interpretation. Fourth, the number of dislocation events was small, so that finding should be interpreted cautiously despite statistical significance. Finally, outcomes managed outside of our health system may have been incompletely captured, and surgeon-driven implant selection may have introduced residual selection bias and confounding; implant selection was surgeon-driven, and bone quality or osteoporosis severity was not consistently documented, limiting our ability to account for these factors in the comparison. Observational shoulder arthroplasty studies are particularly vulnerable to causal overstatement, and the present findings should therefore be interpreted as associations rather than proof of implant effect.^34^ Future comparative studies with more uniform case mix, standardized radiographic assessment, and longer follow-up are needed to clarify whether the observed efficiency and functional differences reflect modularity itself or residual confounding. More tightly matched comparisons accounting for fracture class, bone quality, age, and sex would be particularly valuable in isolating the effect of stem modularity.

## 5 Conclusions

Modular humeral stems were associated with comparable short-term safety, shorter operative time, shorter LOS, higher rates of discharge to home, and higher available ASES scores after shoulder arthroplasty for PHF in this multicenter cohort. However, prosthetic dislocation occurred significantly more frequently in the modular group, and nonrandom differences between cohorts limit causal interpretation. Modular stems appear to be a reasonable option in fracture arthroplasty, but further comparative studies are needed to clarify whether these observed differences reflect modularity itself or residual confounding.

## Informed consent

The requirement for informed consent was waived because of the retrospective study design and use of deidentified data.

## Ethics approval

This retrospective study was approved by the Institutional Review Board (IRB 23-0312). The requirement for informed consent was waived due to the retrospective design and use of deidentified data.

## CRediT authorship contribution statement

Aaron M. Atlas: Conceptualization, Data curation, Formal analysis, Writing – original draft, Writing – review & editing. Shawn J. Geffken: Conceptualization, Data curation, Formal analysis, Writing – original draft, Writing – review & editing. Benjamin Hershfeld: Conceptualization, Data curation, Formal analysis, Writing – original draft, Writing – review & editing. Matthew T. Geiselmann: Conceptualization, Data curation, Formal analysis, Writing – original draft, Writing – review & editing. Anthony Modica: Conceptualization, Data curation, Formal analysis, Writing – original draft, Writing – review & editing. Nabil Khan: Conceptualization, Data curation, Formal analysis, Writing – original draft, Writing – review & editing. Brett Lenart: Conceptualization, Supervision, Writing – review & editing.

## Funding

This research did not receive any specific grant from funding agencies in the public, commercial, or not-for-profit sectors.
