# Corrigendum to “Modular versus non-modular humeral stems in shoulder arthroplasty for proximal humerus fracture: a multicenter retrospective cohort study” [J Orthopaed 77 (2026) 36-41]

**DOI:** 10.1016/j.jor.2026.07.001

**Published:** 2026-07-20

**Authors:** Aaron M. Atlas, Shawn J. Geffken, Matthew T. Geiselmann, Benjamin Hershfeld, Anthony Modica, Randy M. Cohn, Brett A. Lenart

**Affiliations:** a Northwell Health, New Hyde Park, NY, USA; b Department of Orthopaedic Surgery, Northwell Health - Huntington Hospital, 270 Park Ave, Huntington, NY, 11743, USA; c Donald and Barbara Zucker School of Medicine at Hofstra/Northwell, 500 Hofstra Blvd, Hempstead, NY, 11549, USA; d Orlin and Cohen Orthopedic Group, 444 Merrick Road, Lynbrook, NY, 11563, USA

The authors inadvertently omitted Brett A. Lenart from the author list in the original publication. Brett A. Lenart should be added as the final author.

The authors would like to apologize for any inconvenience caused.

